# Healthy Eating Index and Alternate Healthy Eating Index among Haitian Americans and African Americans with and without Type 2 Diabetes

**DOI:** 10.1155/2011/398324

**Published:** 2011-12-08

**Authors:** Fatma G. Huffman, Maurcio De La Cera, Joan A. Vaccaro, Gustavo G. Zarini, Joel Exebio, Deva Gundupalli, Lamya Shaban

**Affiliations:** Department of Dietetics and Nutrition, Robert Stempel College of Public Health and Social Work, Florida International University, Miami, FL 33199, USA

## Abstract

Ethnicities within Black populations have not been distinguished in most nutrition studies. We sought to examine dietary differences between African Americans (AA) and Haitian Americans (HA) with and without type 2 diabetes using the Healthy Eating Index, 2005 (HEI-05), and the Alternate Healthy Eating Index (AHEI). The design was cross-sectional *N* = 471 (225 AA, 246 HA) and recruitment was by community outreach. The eating indices were calculated from data collected with the Harvard food-frequency questionnaire. African Americans had lower HEI-05 scores *β* = −10.9 (−8.67, 13.1); SE = 1.12, *P* < .001 than HA. Haitian American females and AA males had higher AHEI than AA females and HA males, respectively, (*P* = .006) adjusting for age and education. Participants with diabetes had higher adherence to the HEI-05 *β* = 3.90 (1.78, 6.01), SE = 1.08, *P* < .001 and lower adherence to the AHEI *β* = −9.73 (16.3, −3.19), SE = 3.33, *P* = .004, than participants without diabetes. The findings underscore the importance of disaggregating ethnicities and disease state when assessing diet.

## 1. Introduction

The risk for death among people with diabetes is approximately twice that of people without diabetes of similar age groups [1]. The prevalence of diabetes for Black non-Hispanic adults (≥20 years) (14.7%) is nearly twice as compared to the general population (8.3%) [1]. Complications of diabetes may be prevented by adherence to a healthy diet [2]. A national survey reported that Americans consume foods that are high in added sugar and solid fats [3]. Blacks may have lower adherence to the national dietary guidelines than the general population. Furthermore, variations in adherence have been found to differ within ethnicities [4, 5]. African Americans have not met the recommended number of servings from any food group despite their individual perception of their diet, which they considered “good or excellent” [6]. The nutritional patterns of Blacks may account for their higher prevalence of diabetes as compared to White non-Hispanics. 

Nutrition recommendations have been made by the government for the prevention of diabetes and its complications [1] based on evidence from prospective studies regarding the relationships among nutrition, health problems, and diabetes [7–12].The national diet has not matched the dietary guidelines and is poorer for Blacks [3–5]. Currently, two indices: the Healthy Eating Index (HEI) and the Alternate Healthy Eating Index (AHEI), a modified version of the HEI, are available and have been used to assess multiple populations [13–16]. The Dietary Guidelines for Americans (DGAs) are the foundation for nutrition policy and guidance in the USA. In order to measure compliance to the DGAs, the United States Department of Agriculture in partnership with the Center for Nutrition Policy and Promotion created the Healthy Eating Index (HEI) in 1995 and has reevaluated it since then [13]. The HEI-05, the most current version, reflects the 2005 DGAs scoring system to address current dietary needs. The index was constructed using data from a national nutrition survey [14]. The scoring system assesses diet quality as opposed to diet quantity [15]. 

Another equally important dietary model is the Alternate Healthy Eating Index (AHEI), which is a modification of the original HEI. It measures diet quality using nine dietary components and can be used to provide dietary guidance for healthy eating. Some of the features that set the AHEI apart from the original HEI and other indices include attention to fat quality, inclusion of moderate alcohol intake, cereal fiber, red-to-white meat ratio, and duration of multivitamin use [16]. Diet quality studies, evaluated by these indices for Black populations with and without diabetes, are scarce. Therefore, the aim of this study was to assess the relationships among the dietary indices: AHEI, HEI-05; diabetes status; two Black ethnicities: African and Haitian Americans.

## 2. Materials and Methods

### 2.1. Study Population

This study was part of a parent study comparing cardiovascular risk factors of two Black ethnicities with and without type 2 diabetes. The purpose of this cross-sectional study was to compare diet quality of two Black ethnicities, African and Haitian Americans, by diabetes status (type 2 diabetes/no diabetes). Recruitment of the participants was from Miami-Dade and Broward Counties, Florida, by community outreach methods. Letters of invitation outlining the study were mailed to diabetes educators and health professionals, requesting their cooperation in recruiting individuals with type 2 diabetes. Invitational flyers were distributed to all Florida International University (FIU) faculty, staff, and students explaining the research protocol and requesting their assistance in the study. Advertisements were placed in local AA and HA newspapers and principal gathering places along with radio ads on local Creole and AA stations. In addition to community outreach, AAs were mailed invitational flyers from a purchase mailing list from Knowledge Base Marketing, Inc., Richardson, Tex, USA. Upon recruitment of the required type 2 diabetes participants, recruitment of participants without diabetes was initiated by similar methods. 

Since this diet quality study was part of a parent study, the inclusion/exclusion criteria was determined to study indicators of cardiovascular risk (including diet). As such, persons with cardiovascular disease (except those with coronary heart disease) and individuals who had cancer were not excluded. Inclusion criteria consisted of male and female AA and HA with self-reported type 2 diabetes, age ≥35 years, not pregnant or lactating, no thyroid disorders, no coronary heart disease, not undergoing chemo- or radiation therapy, and no major psychiatric disorders. Respondents with diabetes were asked for the age of diagnosis with diabetes and initial treatment modalities (with or without insulin). The participants without diabetes met the same criteria, except they were free of diabetes. A total of 516 participants completed the study. Ten participants had missing values and were therefore excluded. In addition, 35 participants were excluded due to extreme energy intake values (≤500 or ≥5,000 calories per day). The final sample consisted of 471 participants for whom all data were available. The study was approved by the Institutional Review Board of Florida International University. All participants read, understood, and signed an informed consent form in either English or Creole.

### 2.2. Measures

Age, gender, duration of residence in the USA, language preference, education, income, employment status, medication history, family history of diabetes, and coronary heart disease were collected using a sociodemographic questionnaire. Participant's height and weight were measured with the subject standing erect without shoes, and, for weight, wearing light clothes using a SECA clinical scale (SECA Corp, Columbia, MD). Waist circumference to the nearest 0.1 cm was measured horizontally with a nonstretchable measuring tape placed midway between the 12th rib and iliac crest at minimal respiration to determine central obesity. Physical activity was assessed with the Modifiable Activities Questionnaire [17]. This instrument measures physical activity by estimated metabolic cost where the reference value is estimated as a metabolic equivalent (MET) of 1 kcal·kg^−1^·h^−1^ and is multiples of resting metabolic rate of a “typical person” sitting quietly. Participants self-reported their leisure and occupational activities over the past year. Each activity was calculated by the number of months it was performed, times per month, and duration of the activity. The total of the activity was divided by 52 weeks per year to obtain hours/week. The activity was then multiplied by the corresponding MET to obtain Met-h/wk. Finally, all the past years leisure and occupational activities in MET-h/wk were totaled to obtain the participant's physical activity level. Depressive symptoms were measured with the beck depression inventory (BDI) [18]. This is a 21-item self-reported questionnaire that assesses the severity of depressive symptoms. Participants whose scores indicated depression were given their results and advised to discuss them with their doctor.

All dietary variables were collected using the Harvard semiquantitative food frequency questionnaire (FFQ) [19]. Participants self-report consumption of foods and vitamins over the past year, and the instrument categorizes macro- and micronutrients. This questionnaire has been extensively validated and standardized in several multiethnic population-based prospective and cross-sectional studies.

### 2.3. Statistical Analysis

Diet quality from the HEI-05 was calculated from each completed FFQ. The HEI-05 consists of 12 components—total fruit, whole fruit, total vegetables, dark green and orange vegetables and legumes, total grains, whole grains, milk, meat and beans, oils, saturated fat, sodium, and energy from solid fats, alcohol, and added sugars (SoFAAS). Twelve individual components of the HEI-05 represent all of the major food groups found in MyPyramid (an online, interactive counterpart based on the DGAs available to the public). The intakes of foods and nutrients are represented on a density basis, as amounts per 1,000 calories [15]. A minimum score of zero for no intake and a maximum score of either 5 for fruits, vegetables, and grains; 10 for milk and meat/bean products, and 10 for oils was assigned for healthy components. For sugars, saturated fat, and sodium, higher scores were assigned for lower intakes. For SoFFAS, the highest score was 20 (representing ≤20% of energy) and the maximum for saturated fat and sodium was 10 points each. Saturated fat and sodium received a score of 8 for the intake levels that reflect the 2005 and 2010 DGAs less than 10% of energy from saturated fat and 1.1 grams of sodium/1,000 kcal, respectively. Total HEI-05 ranges from 0 to 100 points, and the higher the score, the more the diet complies with the 2005 DGAs, presuming a better diet quality [15]. 

The AHEI score was calculated from each completed FFQ using the AHEI developed by McCullough et al. [16]. Food items listed on the FFQ were assigned to their appropriate food groups. Eight of the nine components of the AHEI contributed zero to ten points to the total score. A score of ten implies that the recommendations were fully met, whereas a score of zero represents the least healthy dietary behavior. Scores between zero and ten were determined proportionally. The items included in the AHEI score were vegetables (servings/day), fruits (servings/day), nuts and soy protein (servings/day), white to red meat ratio, cereal fiber (g/day), trans fatty acids (percent of total energy), polyunsaturated to saturated fatty acid ratio, duration of multivitamin use, and alcohol intake (servings/day). The multivitamin component was the only component that was dichotomized, and a score of 2.5 or 7.5 was given for intake of less than five years or more than five years, respectively. Lastly, all individual component scores were summed for a total AHEI score ranging from 2.5 (least desirable) to 87.5 (most desirable) dietary pattern. 

Summary statistics were calculated for sociodemographic variables, HEI-05, AHEI scores, and their corresponding individual component scores stratified by ethnicity and diabetes status. Student *t*-tests were performed for continuous variables, and Chi-square was conducted for categorical variables. General linear models were performed to examine the associations between the independent variables: ethnicity, diabetes status, and gender with AHEI / HEI-05 scores as the dependent variables. Full models contained the independent variables, 2-way interactions (diabetes status by ethnicity, diabetes status by gender, ethnicity by gender), a 3-way interaction of diabetes status by ethnicity by gender, and potential covariates (sociodemographic, depression symptoms, and depression medications). The following covariates were tested: age, education, waist circumference, physical activity level, kilocalories consumed per day, smoking status, depression score, and antidepression medications (yes/no). Covariates judged to be clinically significant (age, gender) or those with *P*  values ≤ .2 were retained for the final models. Education level was coded as follows: “low education level (< than high-school diploma),” “intermediate (high-school diploma or some college),” and “high education level (college and above).” A two-tailed, *P*  value  of < .05 was considered significant, and IBM SPSS version 18 was used for statistical analyses.

## 3. Results

### 3.1. General Characteristics of the Participants

Descriptive statistics for the study population are listed in Table 1. The mean age was about three years lower for HA than AA (*P* = .003) and about four years lower for participants with as compared to without diabetes (*P* < .001). The ratio of sexes was relatively balanced (52.4% female). Individuals with type 2 diabetes had less education then those without the disease (*P* = .008). African Americans were significantly more educated (*P* < .001) than HA. Approximately 66% of AA had a high-school diploma and/or some college compared to 37.8% of HA. Haitian Americans consumed significantly less calories and had smaller waist circumferences. Physical activity levels were approximately one-third lower than recommended for both AA and HA [20]. There were no differences in smoking by diabetes status; however, nearly 35% of AAs reported being current smokers as compared to less than 7% of HA. Participant with diabetes had lower physical activity levels and higher waist circumference than those without diabetes. Individuals with diabetes were associated with a higher mean BDI score as compared to those without diabetes. There was no significant difference in the BDI scores between ethnicities (*P* = .092); however, more AA (13.8%) reported taking depression medications compared to HA (5.3%) (*P* = .002). 

### 3.2. Healthy Eating Index

Mean HEI-05 scores for African and Haitian Americans and by disease state are shown in Table 2. 

The scoring standards and presentation of this table was replicated from the CNNP Fact Sheet no. 1, 2006 [21], and modified by the adding our data for ethnicity. The mean HEI-05 scores for African and Haitian Americans were 56.3 ± 12.5 and 66.4 ± 11.6 (*P* < .001), respectively. Haitian Americans had significantly higher scores than AA on nine of the twelve components: total fruit (*P* = .006), whole fruit (*P* = .001), total vegetables (*P* = .001), total dark green and orange vegetables (*P* = .001), total grain (*P* = .001), whole grain (*P* = .001), saturated fat (*P* = .001), sodium (*P* = .001), and calories from solid fats, alcohol, and added sugar (SoFAAS) (*P* = .001). 

Unadjusted HEI-05 scores were higher in HA than for AA (*P* < .001) and for persons with as compared to without diabetes (*P* = .001) (Table 2). These relationships were maintained with multivariate adjustments. Disease state (*F*_1,464_ = 12.1, *P* < .001), ethnicity (*F*_1,464_ = 94.7, *P* < .001), gender (*F*_1,464_ = 34.4, *P* < .001), age (*F*_1,464_ = 4.89, *P* = .027), and education (*F*_2,464_ = 7.29, *P* = .001) were associated with HEI-05 (outcome) by the general linear model. Individuals with diabetes were associated with higher HEI-05 scores as compared to those without diabetes (*β* = 3.90 (1.78, 6.01), SE = 1.08, *P* < .001). Being HA (*β* = 10.9 (8.67, 13.1), SE = 1.12, *P* < .001) and female (*β* = 6.19 (4.12, 8.26), SE = 1.05, *P* < .001) were positively associated with HEI-05 score. The HEI-05 score was negatively associated with having less than a college degree (*β* = −6.17 (−9.41,−2.93), SE = 1.65, *P* < .001, *<high school*; *β* = −2.92 (−5.83,−.003), *P* = .05, *at least high school-some college*). This final model for HEI-05 explained 24.3% of the variance of the HEI-05 score (*F*_6,464_ = 26.1, *P* < .001, adj. *R* ^2^ = 0.243).

### 3.3. Alternate Healthy Eating Index

The unadjusted, mean AHEI scores for AA and HA and by diabetes status are shown in Table 3.

The total AHEI score was higher for HA as compared to AA. Haitian Americans had significantly higher scores on several components including vegetables fruit, nuts and soy protein, white to red meat ratio, cereal fiber, trans-fat (lower consumption), and polyunsaturated to saturated fatty acid ratio. In contrast, AA had significantly higher scores for the multivitamin (*P* = .001) and alcohol components (*P* = .003). 

The unadjusted comparison (Student *t*-test) showed no significant differences in the total AHEI score due to diabetes status (*P* = .120) (Table 3). Several significant patterns emerged for the AHEI categories by diabetes status. Participants without diabetes scored higher for consumption of the non-animal source “meat group” (beans, seeds, soya protein), fruit, and polyunsaturated fatty acids to saturated fatty acids (P : S) ratio. Conversely, persons with diabetes scored higher in cereal fiber than persons without diabetes.

Diabetes status (*F*_1,459_ = 5.51, *P* = .019), ethnicity (*F*_1,459_ = 50.8, *P* < .001), the interaction of gender by ethnicity (*F*_1,459_ = 7.77, *P* = .006), and education (*F*_2,459_ = 4.84, *P* = .008) were associated with AHEI (outcome) by the final adjusted general linear model. The associations of ethnicity and education with AHEI followed the same pattern as the HEI-05 whereas the association of diabetes with AHEI was the opposite compared to HEI-05. Individuals with diabetes were associated with lower AHEI scores than those without diabetes (*β* = −9.73 (16.3, −3.19), SE = 3.33, *P* = .004) after final adjustments (ethnicity, gender, ethnicity by gender, age, and education). There was a significant interaction of ethnicity and gender. African American males and HA females had higher AHEI scores than AA females and HA males, respectively, (*P* = .006) adjusting for age and education. The model (*F*_11,459_   = 8.71, *P* < .001) explained 15.3% of the variance of AHEI (adj. *R* ^2^ = 0.153).

## 4. Discussion

We found significant differences in dietary patterns of individuals with diabetes using two indices: HEI-05 and AHEI. Assessing diet by the HEI-05, persons with diabetes were considered healthier eaters than those without type 2 diabetes whereas individuals with type 2 diabetes scored lower than those without diabetes using the AHEI after multivariate adjustments. Calorie intake differences by ethnicity and diabetes status may be a confounder in comparing these indices. Significant difference by diabetes status for HEI-05 and not AHEI may be due, in part, to the construction of these indices. The HEI-05 adjusts for energy intake, while the AHEI does not. Participants with diabetes consumed less calories than those without diabetes. The performance of the HEI-05 may be more suitable for individuals with diabetes since it has a separate category for added fats and sugars. Differences in fat scores between these indices may also make the HEI-05 more suitable for measuring diet quality for persons with diabetes than the AHEI. The AHEI ranks monounsaturated and polyunsaturated fats higher than the HEI-05. Individuals with diabetes may be more inclined to eat a low-fat diet as recommended by the American Heart Association [22] and the American Diabetes Association [2]. 

Several differences in the scoring of these indices may be attributed to differences in disease state. There are major differences in the rating of fat and sugar by the two indices. The AHEI rates alcohol in moderation healthy whereas the HEI-05 scores any alcohol as negative, together in a category with added sugars and solid fats (SoFFAS). Participants with diabetes scored higher in the SoFFAS category (indicating fewer calories from SoFFAS) than those without diabetes. This trend supports a higher HEI-05 for persons with compared to without diabetes. Conversely, the higher P : S ratio for persons without as compared to with diabetes in the AHEI (absent in the HEI-05 index) supports healthier fat type consumption for participants without the disease. Fruit juice is considered a separate category for HEI-05 whereas, in the AHEI, this distinction is not made. Consumption of whole fruit as opposed to fruit juice, generally recommended by diabetes educators, may account for the lower overall fruit consumption of individuals with compared to those without diabetes. This trend was significant only for the AHEI. A low consumption of fruit by Blacks may explain the lack of power to detect a difference in fruit consumption over two categories (juice versus whole fruit). Although persons with diabetes scored higher than those without diabetes in the meat and bean category of the HEI-05, they scored lower in the nuts and soy AHEI category. On the other hand, there was no difference in the ratio of white to red meat AHEI category by diabetes status. Fiber (whole grains on HEI-05 and cereal fiber on AHEI) was one category that participants with as compared to without diabetes scored higher on both indices. Our findings regarding lower consumption of sweets and fruit juices measured by the HEI-05 for participants with diabetes as compared to those without diabetes corroborate with similar finding from a large epidemiological study in multiethnic European populations [23]. The investigators further reported consumption of vegetables, fish, and meat was slightly higher (<10%) for individuals with diabetes as opposed to without diabetes; albeit, they affirmed this difference was small on an absolute scale and may reflect a nutrition education bias for participants with diabetes [23].

There are a number of factors making it difficult to compare our sample to the literature in terms of diet quality. Currently, there are no cutoff points for high adherence for either index. There have been few studies of the performance of either of these indices in populations with type 2 diabetes or Black populations. Furthermore, there are no studies to our knowledge that compared individuals with type 2 diabetes or Black populations to their counterparts. Evaluation of the performance of these indices is further limited by the types of studies available. Measures for the performance of either index have been morbidity and mortality health outcomes, for the majority of studies.

Relative risk of new cases of type 2 diabetes for men (*N* = 41,615) from the Health Professionals Follow-up Study after a 20-year followup were lower for those with higher AHEI but the relationship was not significant for HEI-05, after multivariate adjustments (age, obesity, smoking, coffee intake, total energy, family history of diabetes, and physical activity) [24]. Similarly, the relative risk for type 2 diabetes for 18 years in women from the Nurses' Health Study (*N* = 80,029) was lower for higher AHEI scores after multivariate adjustment (age, energy intake, smoking obesity, family history, menopausal status, and postmenopausal hormone use); however, the investigators did not measure the HEI-05 [8]. The HEI-05 may be useful as a screening tool for persons at risk for diabetes. Data from 11 prospective studies in a meta-analysis (*N* = 210,819) revealed that individuals with the highest quartile intake of sugar-sweetened beverages (1-2 servings per day) had a 26% greater risk of developing type 2 diabetes than those with the lowest quartile intake (none or <1 serving per month) [25]. 

A dietary pattern of high vegetable and fruit intake and low red meat and fried food consumption maintained over time was associated with 5 kg less weight gain (5th quintile compared to 1st quintile) after 14 years in subgroup of Black women (*n* = 12,736 from *N* = 41,351 tested) from the Black Women's Health Study [26]. For our study, gender interacted with ethnicity across diabetes status for the AHEI; however, gender was independent of ethnicity using the HEI-05. African American women and HA males scored lower on the AHEI than their counterparts. Higher AHEI scores have been associated with a lower risk for type 2 diabetes in large prospective studies of women [8] and the “prudent pattern” (a diet similar to AHEI) for men [12]. These findings suggest AA women and HA men would profit from dietary counseling to lower the risk for developing type 2 diabetes (for those without diabetes) and to help reduce diabetes complications (for those with diabetes).

Haitian Americans had a healthier diet compared to AA and showed marked differences in certain components such as SoFAAS in this study. Findings should not be interpreted as HA having a healthy diet. In fact, some of the individual component scores from both indices remained low in HA when compared to national nutrition surveys [27]. African Americans had a higher consumption of processed and red meat (bacon, cold cuts, and beef) as compared to HA. In contrast, and as reflected by their higher scores, HA had higher intakes of fish, chicken, most fruits and vegetables, nuts, and soy.

The mean scores for both indices were lower than optimal for both HA and AA and by disease state (approximately two-thirds of the HEI and one-half of the AHEI). Poor dietary quality of these Black ethnicities may be indicative of dietary issues specific to their population. Assessment of eating by dietary indices may reveal dietary issues specific to a population and can ultimately result in positive health outcomes [28]. National Centers for Health Statistics have combined HA in the category “Blacks” [29]. There are few studies that investigated dietary intake of HA by diabetes status; however, a recent study compared diabetes care outcomes among HA, AA, and White non-Hispanics (WNH) with diabetes [30]. The investigators reported HA had worse glycemic control as compared to AA and WNH and recommend future interventions target the Haitian culture [30]. 

Regarding differences between HA and AA, findings from our study are consistent with those from Lancaster et al. [4] in which Blacks born outside the USA had higher intakes of fruits, vegetables, and fiber and lower intakes of fat and added sugars than those born in the USA. Similarly, another study found that foreign born African Caribbean participants had better diet scores and lower BMIs than USA born AA males but not females [5]. Over ten distinct dietary patterns for *N* = 763 AA residing in Florida were found by cluster analysis [6]. The results suggested that there is no “typical” eating pattern for AA and that these patterns varied by gender [6].

There are several potential limitations associated with our study. First, due to the cross-sectional nature of the study, causality cannot be inferred from the results. Second, there was a lack of a consensus for the calculation of the individual components from the Harvard's FFQ [31, 32]. On the other hand, choosing the Harvard FFQ may better reflect intake of foods consumed infrequently or seasonally since it has been validated to estimate food and nutrient intake relative to long-term diet records [33]. Finally, the study population may not be representative of HA and AA since individuals choosing to participate may differ from the general population. 

Despite these caveats, this study has a number of strengths. (1) No published studies have been conducted comparing the HEI-05, AHEI, and their relationships to type 2 diabetes outcomes in AA and HA. (2) Most studies have used a 24-hour recall rather than a standardized food frequency questionnaire for the calculation of the HEI-05 and the AHEI. (3) Our study constitutes the first study to consider dietary differences between two Black ethnicities, with and without type 2 diabetes, evaluated through two distinct eating indices. In agreement with the recommendation of several studies [4, 5], future studies of diet and health should be conducted with respect to the cultural and gender differences within the Black population. Moreover, disease state needs to be taken into account when choosing an appropriate diet index.

## 5. Conclusions

African Americans had less favorable dietary patterns than Haitian Americans. This finding strengthens the need to consider ethnicity in health studies, despite the fact that ethnic differences have been commonly overlooked for the Black populations living in the United States. Participants with type 2 diabetes were more adherent to the HEI-05 and less adherent to the AHEI as compared to participants without diabetes. Ethnicity and gender differences for dietary patterns assessed by AHEI suggest AA females and HA males may be at risk for cardiovascular disease, independent of diabetes status. These findings reinforce the need for culturally sensitive, dietary counseling aimed at primary and secondary prevention of type 2 diabetes.

## Figures and Tables

**Table 1:** General characteristics of the participants by ethnicity and diabetes status.

Variables	Ethnicity			Diabetes status		
	AA (*n* = 225)	HA (*n* = 246)	*P*	Without T2D (*n* = 218)	With T2D (*n* = 253)	*P*
Age (years)	53.0 ± 9.7	55.8 ± 10.6	.003	52.4 ± 9.9	56.3 ± 10.3	<.001
Gender (female) *n* (%)	118 (52.4)	129 (52.4)	.999	111 (44.9)	136 (55.1)	.539
Waist circumference (cm)	108 ± 18	98 ± 12	<.001	98.5 ± 13.6	106.8 ±16.7	<.001
Without T2D (*n *= 218)	101 ± 14	96 ±13	.009	—	—	
With T2D (*n *= 253)	114 ± 18	100 ± 12	<.001	—	—	
Education level *n* (%)	—	—	.001	—	—	.008
< High school graduate	36 (16.0)	114 (46.3)	—	57 (26.1)	93 (36.8)	
High school or some college	148 (66.2)	93 (37.8)	—	114 (52.3)	128 (50.6)	
College degree/beyond	40 (17.8)	39 (15.9)	—	47 (21.6)	32 (12.6)	
Smoking (yes) *n *(%)	78 (34.7)	16 (6.5)	.001	42 (19.3)	52 (20.6)	.727
Total calories/day (Kcal)	2142 ± 1016	1751 ± 912	.001	2037 ±1022	1852 ± 939	.043
HEI-05 scores	56.3 ± 12.5	66.4 ± 11.6	<.001	59.5 ± 13.3	63.4 ±12.5	.001
AHEI scores	42.4 ± 12.6	51.4 ± 12.6	.001	48.2 (14)	46.2 (13)	.120
Physical activity (MET-h/wk)	51 ± 87	45 ± 67	.393	60.7 ± 89	36.8 ± 62	.001
BDI scores	8.2 ± 8.8	9.5 ± 8.1	.092	7.3 ± 7.7	10.3 ± 8.8	<.001
Depression meds (yes) *n* (%)	31 (13.8)	13 (5.3)	.002	17(7.8)	27 (10.7)	.285

AA: African American; AHEI: Alternate Healthy Eating Index; HA: Haitian American; T2D: type 2 diabetes; HEI-05: Healthy Eating Index, 2005 MET-h/wk: metabolic equivalent of leisure and occupational activities over the year and converted to hours per week 1-MET is an estimated ratio of a working metabolic rate to a standard resting metabolic rate (1 Kcal kg^−1^ hr^−1^) for an average individual; BDI: beck depression Inventory.

*Notes*: *P* values reported are based on the Student *t* -test or chi-square are unadjusted. A *P* value of <.05 (two-tailed) was considered significant.

**Table 2:** Healthy Eating Index 2005 (HEI-05) score components and total scores^a^ by ethnicity and diabetes status.

Component	Criteria			Ethnicity			Diabetes status		
	Score Ranges	Standard for minimum score	Standard for maximum score	AA	HA	*P*	Without T2D	With T2D	*P*
Total fruit (includes 100% fruit juice)	0–5	No fruit	≥0.8 cup/ 1000 kcal	3.9 ± 1.5	4.3 ± 1.3	.006	4.2 ± 1.3	4.0 ± 1.3	.076
Whole fruit (excluding fruit juice)	0–5	No whole fruit	≥0.4 cups/ 1000 kcal	3.6 ± 1.6	4.2 ± 1.4	<.001	4.0 ± 1.5	3.9 ± 1.5	.579
Total vegetables	0–5	No vegetables	≥1.1 cups/ 1000 Kcal	3.8 ±1.2	4.6 ± 0.9	<.001	4.1 ± 1.1	4.3 ± 1.1	.160
Dark-green/ orange vegetables/ legumes^b^	0–5	No dark-green/ orange vegetables/ legumes	≥0.4 cup/ 1000 kcal	2.2 ± 1.5	3.5 ± 1.6	<.001	2.9 ± 1.7	2.9 ± 1.7	.752
Total grains	0–5	No grains	≥3.0 cups/ 1000 kcal	3.5 ± 1.1	3.9 ± 1.1	<.001	3.6 ± 1.1	3.8 ± 1.1	.044
Whole grains	0–5	No whole grains	≥1.5 oz/ 1000 kcal	1.7 ± 1.2	2.5 ± 1.6	<.001	1.8 ± 1.3	2.3 ± 1.6	<.001
Milk^c^	0–10	No milk	≥1.3 cups/ 1000 kcal	4.2 ± 2.7	4.1 ± 3.0	.905	3.7 ± 2.8	4.6 ± 2.9	.001
Meat and beans	0–10	No meat or beans	≥2.5 oz/ 1000 kcal	5.8 ± 1.3	5.6 ± 1.4	.337	5.4 ±1.3	5.9 ± 1.3	<.001
Oils^d^	0–10	No oil	≥12 g/ 1000 kcal	6.7 ± 4.7	6.3 ± 4.8	.307	6.4 ± 4.8	6.6 ± 4.8	.676
Saturated fat^e^	0–10	≥ 15 % of energy	≤7% of energy	6.4 ± 2.5	8.3 ± 2.1	<.001	7.6 ± 2.4	7.2 ± 2.5	.080
Sodium	0–10	≥ 2.0 g/1000 kcal	≥0.7 g/ 1000 kcal	4.0 ± 3.1	5.7 ± 3.1	<.001	5.0 ± 3.2	4.8 ± 3.2	.530
SoFFAS	0–20	≥ 50% of energy	≤20% of energy	10.5 ± 6.8	13.4 ± 6.8	<.001	10.8 ± 6.8	13.1 ± 7.8	<.001
Total HEI-05 score	0 (worst) to 100 (best)			56.3 ±12.5	66.4 ±11.6	<.001	59.5 ±13.3	63.4±12.5	.001

AA: African American; HA: Haitian American; T2D: type 2 diabetes; SoFFAS: calories from solid fats, alcohol, and added sugar.

^a^Intakes between the minimum and maximum levels were scored proportionally, except for saturated fat and sodium (see note e).

^b^Legumes counted as vegetables only after meat and beans standard is met.

^c^Includes all milk products, such as fluid milk, yogurt, and cheese.

^d^Includes nonhydrogenated vegetable oils and oils in fish, nuts, and seeds.

^e^Saturated fat and sodium received a score of 8 for the intake levels that reflect the 2005 DGAs. <10% of energy from saturated fat, and 1.1 grams of sodium/1000 kcal, respectively. *P*-values are based on the Student *t*-test and are unadjusted. A *P* value of <.05 (two-tailed) was considered significant. Scores are means ± SD.

**Table 3:** Alternate Healthy Eating Index (AHEI) score components by ethnicity and diabetes status.

Component	Criteria		Ethnicity			Diabetes status		
	Minimum score of 0_a_	Maximum score of 10_a_	AA	HA	*P*	Without diabetes	With type 2 diabetes	*P*
Vegetables (s/d)	0	5	5.5 ± 3.0	7.5 ± 2.9	.001	6.5 ± 3.1	6.5 ± 3.1	.980
Fruits (s/d)	0	4	5.4 ± 3.1	6.1 ± 3.3	.013	6.2 ± 3.3	5.4 ± 3.2	.004
Nuts and soy proteins (s/d)	0	1	3.5 ± 3.4	4.4 ± 3.7	.003	4.4 ± 3.5	3.6 ± 3.6	.003
Ratio of white to red meat	0	4	3.8 ± 2.9	7.3 ± 3.3	.001	5.7 ± 3.5	5.6 ± 3.5	.913
Cereal fiber (g/d)	0	15	4.7 ± 3.6	5.5 ± 3.7	.014	4.7 ± 3.6	5.5 ± 3.8	.016
Trans fat (% of total energy)	≥4	≤.5	8.2 ± 1.2	9.4 ± 0.8	.001	8.9 ± 1.1	8.7 ± 1.1	.151
P : S Fat	≤.1	≥1	6.5 ± 1.9	7.5 ± 2.1	.001	7.2 ± 2.1	6.8 ± 2.0	.041
Duration of MVI use^b^	<5 years	≥5 years	3.5 ± 2.0	2.9 ± 1.4	.001	3.3 ± 1.8	3.1 ± 1.6	.243
Alcohol (s/d)^c^	M: 0 or >3.5 W: 0 or >2.5	M:1.5–2.5 W: 0.5–1.5	1.5 ± 2.7	0.8 ± 1.9	.003	1.3 ± 2.6	0.9 ± 2.0	.057
Total AHEI score	2.5	87.5	42.4 ± 12.6	51.4 ± 12.6	.001	48.2 ± 14	46.2 ± 13	.120

AHEI: Alternate Healthy Eating Index; AA: African American; HA: Haitian American; MVI: multivitamin intake; P : S Fat: polyunsaturated fatty acids to saturated fatty acids ratio; s/d: servings per day; M: men; W: women.

^a^Intermediate intakes were scored proportionally.

^b^For multivitamins, the minimum score was 2.5 and the maximum score was 7.5 (dichotomized).

^c^Beer, wine, and liquor.

*Note*: Table adapted from USDA Center for Nutrition Policy and Promotion. *P* values are based on the Student *t*-test and are unadjusted. Scores are means ± SD.
